# Attitudes and gaps in knowledge of the diagnosis, treatment, and psychopathology of eating disorders among different health professionals

**DOI:** 10.1186/s40337-024-01053-5

**Published:** 2024-06-28

**Authors:** Alessio Maria Monteleone, Marco Carfagno, Eugenia Barone, Giammarco Cascino, Armando Pitocco, Carlotta Brandi, Lorenzo Landolfi, Claudia Toni, Gaia Sampogna, Andrea Fiorillo

**Affiliations:** 1 Department of Psychiatry, University of Campania “Luigi Vanvitelli”, Largo Madonna delle Grazie, Naples, 80138 Italy; 2 Department of Medicine, Surgery and Dentistry ’Scuola Medica Salernitana’, Section of Neurosciences, University of Salerno, Salerno, Italy

**Keywords:** Eating disorders, Anorexia nervosa, Stigma, Education, Health professional

## Abstract

**Background:**

Health professionals from different specialties in medical and psychological areas play an important role in diagnosis and treatment of eating disorders (EDs). This study aimed to identify gaps in knowledge about the diagnosis, etiology, and management of EDs and to assess health professionals’ attitudes towards these illnesses.

**Methods:**

A new questionnaire was developed and validated. Residents and consultants working in disciplines involved in the management of EDs (namely, internal medicine, general practitioners, psychiatric area, psychological area, and surgical area) completed the questionnaire. Knowledge and attitudes were compared among the study groups through one-way ANCOVA and chi-square tests.

**Results:**

The final version of the questionnaire consisted of 54 items assessing the following areas: stigma, treatment, physical complications, diagnosis, and aetiopathogenesis of EDs. For all health professionals the area of most deficiency was the aetiopathogenesis, while the best one was the management of physical complications. All medical professionals showed less knowledge than psychiatrists in terms of etiology, diagnosis, and treatment of EDs. A lack of knowledge about evidence-based psychotherapies, general psychopathology, and family members’ role in the management of EDs emerged among all health professionals. Stigma was found among non-mental health professionals who considered these patients to be different from others and responsible for their abnormal eating behaviors.

**Conclusions:**

Clarifying the health professionals’ specific gaps occurring in the knowledge of EDs and in the attitudes towards these individuals may inform educational programs to improve early detection and management of EDs.

## Abstract (plain language summary)

This article investigated attitudes towards individuals with eating disorders (EDs) and knowledge of the diagnosis and management of these illnesses among health professionals. A gap in the knowledge of diagnosis, aetiopathogenesis, and treatment emerged above all in non-mental health professionals. Psychotherapy effectiveness and the role of family members in the therapeutic process were not sufficiently acknowledged, and general psychological factors contributing to the onset of EDs were not recognized. Impaired attitudes were primarily observed among surgeons, although all health professionals considered these disorders as distinct from others and viewed these individuals as responsible for their abnormal eating behaviors. These findings outline a type of stigma towards EDs that is associated with health professionals and may impair early diagnosis and recovery. Educational programs should aim to provide continuous education to update and improve the knowledge of EDs among health professionals.

## Background

Eating disorders (EDs) are complex psychiatric disorders with high rate of psychiatric comorbidities, physical complications, and social impairment [1]. Although the highly prevalent among adolescents and young adults, EDs can occur across all ages, as well as ethnicities and social levels [2]. Despite a decreasing mortality rate over time [3], AN still has the highest rate of mortality among psychiatric disorders [4], with a high rate of chronicity and inadequate recovery [3]. Children and adolescents have better outcomes and a lower rate of chronicity than adults across and within EDs [3]. Illness duration is a negative prognostic factor [5], and a staging model of the illness has been suggested [6]: while ED progresses, several psychosocial and neurobiological factors make the persons resistant to treatment, promoting the persistence of the illness [6]. This supports the need for patients with EDs to be identified early and moved to specialist care as quickly as possible. The guidelines suggest that treatment should be provided at the first detection [7].

Despite this recommendation, only 20–25% of individuals access specialist care for their symptoms [8]. This problem is favored by the complexity of EDs, which require a multidisciplinary approach to address physical, behavioral, and psychosocial problems [9]. Further barriers include lack of awareness of the illness, mental health-related stigma, family knowledge about the disorder, fragmented youth mental health care and organization of the pathways that lead individuals to seek care for their ED [8, 10]. A recent European multicenter study showed that the involvement of many health professionals and the occurrence of affective symptoms (i.e., anxious and depressive symptoms) delay access to specialist ED units [11]. In addition to mental health professionals, general practitioners play a central role in early diagnosis and treatment promotion for EDs [11]. Individuals with EDs consult general practitioners more frequently than those without EDs in the five years before receiving the ED diagnosis [12] and have a high rate of access to mental and physical health services [13]. Involving non-mental health professionals is important both for primary care diagnosis and for treatment: a higher number of specialist treatment components are associated with higher rates of recovery, and a multidisciplinary treatment approach predicts better outcomes in EDs [3].

This claims that an accurate knowledge of ED assessment and treatment is essential among health professionals. Unfortunately, clinicians who are not not specialized in EDs did not show better knowledge about EDs than students or individuals with EDs [14]. Clinicians claim that they lack the necessary skills to intervene when treating individuals with EDs [15] and report stress and negative reactions such as hopelessness, lack of competence, and worry that are associated with their stigmatizing beliefs, inexperience, and gender [16, 17]. Their lack of confidence in helping individuals with EDs may be due to their lack of necessary training [18]: this may result in treatment delays or inappropriate management. Psychiatrists also felt confident in diagnosing EDs, while they reported low confidence in the management of EDs [19].

Given this background, it is important to investigate the knowledge gap between health professionals, considering the differences among clinical specialists. By addressing this gap, educational training could be improved. The primary objective of this study was to identify specific areas where health professionals have inadequate knowledge about EDs, including stigma beliefs, assessment procedures, managing physical complications, treatment options, and risk factors. The second objective of the study was to examine whether there are differences in knowledge and attitudes regarding EDs among health professionals involved in ED management (namely, mental health, primary care, internal medicine, surgical area).

## Methods

### Measures and procedure

The study included three steps. First, a panel composed of four researchers and clinicians of varying seniority ranging from residents to consultants and working in the ED field generated the items of the questionnaire. The items were developed from the *Questionario sulle Opinioni degli Italiani riguardo a.i. disturbi mentali* [20], which was validated for the assessment of clinicians’ knowledge and stigma towards serious mental illnesses. The items were modified considering the Italian guidelines for diagnosis and management of EDs [21], the DSM-5 ED diagnostic criteria [22] and research team working experience. The first version of the questionnaire consisted of 73 items. In the second stage, the original items were reviewed by three expert psychiatrists and were critically discussed, reviewed and, when necessary, rewritten to improve clarity and readability.

The next steps involved recruiting people who were given the pre-final version of the questionnaire, the analysis of the main psychometric properties of the instrument, and the finalization of the questionnaire.

The study was open to participants who worked at University of Campania L. Vanvitelli and met the following inclusion criteria: (a) working as a resident or a consultant in one of the following disciplines: psychiatry, child neuropsychiatry, pediatrics, cardiology, dermatology, endocrinology, gastroenterology, internal medicine, nephrology, neurology, general surgery, gynecology, orthopedics, urology; b) being a psychologist; and c) being a general practitioner working as supervisor for medical students. A convenience sampling method was used to enroll participants, who received an email that included a link to an online questionnaire. The minimum number of respondents was 365, given that validating a questionnaire requires 5 respondents for each item [23].

Participants were more frequently female (58%), with a mean age of 34.2 (± 10.2) yrs. 12% were specialized in a surgical discipline (i.e., general surgeons, gynecologists, orthopedics, and urologists), 32.6% were included in the general practitioner area (i.e., general practitioners and pediatricians), 29% were specialized in a discipline belonging to the internal medicine area (i.e., cardiologists, dermatologists, endocrinologists, gastroenterologists, internists, nephrologists, and neurologists), 17% were included in the psychiatric area (i.e., psychiatrists and child neuropsychiatrists) and 8% were included in the psychological area (i.e., psychologists).

Participants filled in the preliminary version of the questionnaire and provided a rating of importance and appropriateness for each item on a 10-level scale (1 = item not important at all; 10 = item very important). They were asked to provide dichotomous (i.e., yes, no), categorical (i.e., yes always, yes sometimes, rarely, never), or multiple answers. A full (namely, 1 point) score was assigned to correct answers. Twenty-five participants were asked to complete the questionnaire after one week to assess the test–retest reliability. The reasons for discrepancies in the test–retest group were explored through an ad hoc schedule and discussed among researchers. Ethics approval was not required for this kind of survey as per local legislation and national guidelines.

### Statistical analysis

The face validity of the items was explored by means of the ratings on the 1–10 appropriateness scale. Pearson’s rho coefficient was used to evaluate the variance of the responses. The test–retest reliability of each item was analyzed through Cohen’s kappa coefficient. Cronbach’s alpha analysis was used to group single items into the hypothesized subscales (content validity).

The rate of correct answers in the entire sample was calculated. A one-way ANCOVA followed by a post hoc Tukey’s test was conducted to compare the general score and those of each assessed topic (i.e., A, B, C, D, E) among the study groups (i.e., surgical area, internal medicine, general practitioners, psychiatric area and psychological area) controlling for age. The topic scores were calculated as the sum of each item included in that topic area.

The chi-square test was performed to compare the rate of correct answers among the study groups. To explore the differences in terms of stigma and deficit of knowledge, the chi-square test was performed only for items with a frequency of correct answers below 67% in the entire sample. A post hoc test with Bonferroni corrections was performed to examine the differences between study groups. The level of significance was set at *p* < .05. All analyses were performed using JASP software [24].

## Results

The final study sample consisted of 405 participants. Participants compiled the prefinal version of the questionnaire consisting of 73 items. From this list, 7 items rated as not important or relevant (i.e., with an appropriateness rating < 6.0) were eliminated, and 12 items were deleted due to low reliability (7 items with Cohen’s kappa coefficient < 0.60; 5 items with Pearson’s rho coefficient < 0.7). The final version of the questionnaire consisted of 54 items including dichotomous and multiple-choice questions scoring from 0 (totally wrong) and 1 (totally correct), which could be grouped into five categories based on the topic, i.e., stigma (A score), treatment (B score), physical complications (C score), diagnosis (D score), aetiopathogenesis (E score), and general knowledge (Total score). The items referred to an example of ED.

The results of the one-way ANCOVA are reported in Table 1.

**Table 1:** Comparison of assessed topics among health professional areas

Topic	Gen (mean ± SD)	Med (mean ± SD)	Psyl (mean ± SD)	Psyt (mean ± SD)	Surg (mean ± SD)	ANCOVA
Stigma	7.583 ± 1.683	7.534 ± 1.451	7.343 ± 1.168	7.831 ± 1.483	6.724 ± 2.199	Age F(1,399) = 11.756 *p* < .001
						Study groups F(4,399) = 3.008 *p* = .018
Treatment	7.368 ± 1.266	7.327 ± 1.112	8.064 ± 1.037	8.030 ± 1.032	7.174 ± 1.407	Age F(1,399) = 10.14 *p* = .002
						Study groups F(4,399) = 8.065 *p* < .001
Complications	7.153 ± 1.147	7.316 ± 0.987	5.557 ± 1.498	7.817 ± 0.950	7.296 ± 1.040	Age F(1,399) = 1.659 *p* = .198
						Study groups F(4,399) = 25.167 *p* < .001
Diagnosis	7.89 ± 1.429	7.898 ± 1.347	7.486 ± 1.353	8.761 ± 1.281	7.429 ± 1.851	Age F(1,399) = 44.055 *p* < .001
						Study groups F(4,399) = 7.825 *p* < .001
Aetiopathogenesis	7.848 ± 1.884	8.051 ± 1.894	8.314 ± 1.952	8.915 ± 2.103	7.714 ± 2.062	Age F(1,399) = 4.509 *p* = .034
						Study groups F(4,399) = 4.295 *p* = .002
Total	37.84 ± 4.598	38.125 ± 4.128	36.764 ± 3.939	41.354 ± 4.826	36.338 ± 6.257	Age F(1,399) = 27.414 *p* < .001
						Study groups F(4,399) = 10.531 *p* < .001

Gen = general practitioner area; Med = internal medicine area; Psyl = psychological area; Psyt = psychiatric area; Surg = surgical area

When the A score (Cronbach’s α = 0.48) was entered in the model as dependent variable, the post hoc analysis showed a significant difference in terms of stigma between the surgical area and psychiatric area (*p* = .007), with psychiatrists reporting lower levels of stigma. Regarding the B score (Cronbach’s α = 0.42), the post hoc analysis indicated a significant difference in the knowledge of treatment of EDs, with a better knowledge found in the psychiatric area compared to surgical (*p* = .003), internal medicine (*p* < .001) and general practitioner (*p* < .001) areas. Psychologists also showed better knowledge of treatment compared to surgical (*p* = .006), internal medicine (*p* = .003) and general practitioner areas (*p* = .005). When the C score (Cronbach’s α = 0.36) was entered in the model as dependent variable, the post hoc analysis showed a significant difference in terms of knowledge of physical complications between psychiatric and all other areas, with psychiatrists reporting a better understanding and management of complications compared to general practitioner (*p* < .001), internal medicine (*p* = .022), and psychological (*p* < .001) areas. In contrast, psychologists showed lower levels of expertise on complications compared to surgical (*p* < .001), general practitioner (*p* < .001), and internal medicine (*p* < .001) areas. When post hoc analysis was conducted for the D score (Cronbach’s α = 0.47), a significant difference in the level of expertise in the diagnosis of EDs has emerged, with psychiatrists showing better knowledge than health professionals included in surgical (*p* < .001), general practitioner (*p* < .001), internal medicine (*p* < .001), and psychological (*p* = .001) areas. The post hoc analysis computed for the E score (Cronbach’s α = 0.5) revealed a significant difference in the level of expertise in the aetiopathogenesis of EDs, with psychiatric area showing better knowledge compared to surgical (*p* = .02), internal medicine (*p* = .029), and general practitioner (*p* = .002) areas. When the total score (Cronbach’s α = 0.71) was entered in the model as dependent variable, the post hoc analysis revealed a significant difference in terms of general knowledge of EDs between psychiatric and all other areas, with psychiatrics showing the best results (*p* < .001).

The rate of correct answers for each item in the entire sample is reported in Table 2.

**Table 2:** Topics of the questionnaire with frequency of right answers for each item in the all sample. The items refer to the description of the following clinical case.In certain periods of their lives, some individuals think or say to be overweight, are dissatisfied with their bodies, restrict food intake, and overthink about food or deny the evidence that they are losing weight. Their behaviours sometimes differ from those observed in most people: they prefer eating alone or, when they think to have failed with their eating patterns and rules or to have lost control on food intake, can self-induce vomiting, abuse laxatives, or do excessive and compulsive physical exercise

ITEM		Frequency of right answers (%)
*Stigma*		
2	May a person with symptoms described above suffer from?	99.5
26	Are diseases such as those described above like any other disease (e.g., diabetes, heart disease, etc.)?	54.2
27	Are diseases such as those described above like any other mental disorder (*e.g., depression, schizophrenia, anxiety*)?	67.7
28	Is it easy to notice if a person has ever had disorders such as those described above?	46.1
29	Do successful people rarely suffer from disorders such as those described above?	84.7
31	Should people with disorders such as those described above have not children?	91.4
33	Should the National Health System spend more money for the care of people with problems such as those described above?	69
40	Is it possible to diagnose a disorder such as those described above through the person’s appearance?	60.8
41	Do people with disorders such as those described above suffer from a mental disorder?	79.3
42	Have people with disorders such as those described above chosen to behave in that way?	64.3
*Treatment*		
4–5	Do you think that psychotropic drugs are effective in the treatment of disorders such as those described above? *If not*, which is the most effective treatment for these disorders?	94.1
6	What treatments should be prescribed to people with disorders such as those described above?	98.3
7	Do you think a multidisciplinary team approach could be useful for people with disorders such as those described above?	89.7
8	If so, which health professionals should be included in that team?	95.1
9	Do you think psychotherapy could help people with disorders such as those described above?	60.1
10	Do you think there are specific evidence-based psychotherapies for disorders such as those described above?	34.5
11	Do you think that a nutritional intervention is the first step to help people with disorders such as those described above?	40.4
12	In which hospital setting patients with disorders such as those described above should be treated?	88.7
32	Should family members be included in the treatment of people with disorders such as those described above?	49.5
39	May patients suffering from a disorder such as those described above and comorbid diabetes be treated with insulin?	13.1
54	Which are the effects of psychotherapy in disorders such as those described above?	70.9
*Physical complications*		
13	May people with disorders such as those described above develop not severe medical complications, i.e., not life-threatening?	76.1
14	In the presence of underweight, which are the most frequent physical complications?	68.2
15	Would you prescribe blood tests to a person with disorders such as those described above to diagnose medical complications?	86
16	Which of these blood tests would you prescribe for a person with disorders such as those described above?	95.8
17	Which of these medical tests could be useful for a person with disorders such as those described above?	64.5
19	Is it important to measure serum electrolytes in people with disorders such as those described above?	89.2
20	If a person with disorders such as those described above has leucopenia, this is most frequently an indication of …	78.8
21	If a person with disorders such as those described above and underweight has heart rate at 52 beats per minute, it is most frequently an indication of …	74.6
23	Is estroprogestinic replacement therapy always indicated in patients with disorders such as those described above and amenorrhea?	76.8
*Diagnosis*		
1	May a person with the symptoms of the disorder described above suffer from one of the following disorders?	95.3
3	If yes, which health professional should treat a person with the symptoms of the disorder described above?	86.9
18	May normal blood tests allow us to rule out a disorder such as those described above?	42.4
22	Is amenorrhea a mandatory diagnostic criterion for disorders such as those described above?	39.4
24	Should people with disorders such as those described above be clearly informed by their doctors about their diagnosis?	87.9
25	Should relatives of those people be clearly informed by the doctors about the patient’s diagnosis?	23.9
35	What does “binge-eating” mean?	85.2
36	What does “compensatory behavior”’ mean?	94.6
37	What does “restrictive behavior” mean?	98.5
38	May achieving a normal body weight be enough to recover from disorders such as those described above?	70.7
53	At what age disorders such as those described above most frequently occur?	99
*Aetiopathogenesis*		
30	Is there any evidence that the behavior of family members is always the cause of disorders such as those described above?	32.5
34	Compared to the general population, is an individual with a family member suffering from a disorder such as those described above more likely to develop the disorder?	67
43	Do disorders such as those described above affect only women?	90.9
44	Does excessive vanity contribute to the onset of disorders such as those described above?	89.2
45	Does low self-esteem contribute to the onset of disorders such as those described above?	45.1
46	Does pathological perfectionism contribute to the onset of disorders such as those described above?	45.8
47	Does excessive sensitivity to external judgment contribute to the onset of disorders such as those described above?	53.9
48	Does drug abuse contribute to the onset of disorders such as those described above?	62.3
49	Does friendship with wrong or untrustworthy people contribute to the onset of disorders such as those described above?	60.1
50	Do beauty models promoted by the mass media contribute to the onset of disorders such as those described above?	88.9
51	Does dieting contribute to the onset of disorders such as those described above?	78.3
52	Do stressful life events (breaking up with a partner, overwork, unemployment, birth of a child) contribute to the onset of disorders such as those described above?	97.3

Rates of errors were compared among health professional areas through chi-square tests: the results are shown in Table 3. Significant results were found for the following items: psychotherapy intervention could help people with EDs (item 9); there are psychotherapy interventions specifically validated for EDs (item 10); nutritional intervention is the first step to help people with EDs (item 11); instrumental examinations useful to evaluate physical complications (item 17); amenorrhea is a mandatory diagnostic criterion (item 22); it is easy to perceive if a person has ever had an ED (item 28); an individual with a family member suffering from EDs is more likely to develop the disorder (item 34); EDs can be due to pathological perfectionism (item 46); EDs can be due to drug abuse (item 48); and EDs can be due to friendship with wrong or untrustworthy people (item 49).

**Table 3:** Comparison of error rates among health professional areas

Item	Chi Value	*p*	Post-hoc test with Bonferroni correction
9	27.815	0.000	Surg, Med, Gen > Psyl Surg > Psyt
10	19.511	0.002	Med > Psyl
11	14.453	0.013	Surg, Med, Gen > Psyl
17	19.088	0.002	Psyl > All areas
18	3.273	0.658	
22	44.469	0.000	All areas > Psyt
25	2.633	0.756	
26	7.667	0.176	
28	16.283	0.006	Surg > Med
30	9.695	0.084	
32	3.954	0.556	
34	14.347	0.014	Med > Psyt
39	6.068	0.300	
40	6.821	0.234	
42	3.725	0.542	
45	4.787	0.442	
46	12.089	0.034	
47	3.121	0.681	
48	21.849	0.001	Surg, Gen > Psyl
49	25.983	0.000	Gen > Psyt, Psyl

Gen = general practitioner area; Med = internal medicine area; Psyl = psychological area; Psyt = psychiatric area; Surg = surgical area

## Discussion

Knowledge and stigma towards EDs among health professionals were assessed in this study. All health professionals experienced difficulties with ED diagnosis and management compared to psychiatrists, while only surgeons displayed heightened stigma compared to psychiatrists. While physical complications management was the area of greatest expertise for health professionals, knowledge of risk factors was the area with the greatest deficiency. Better knowledge and less stigma were linked to higher participant age. In each study group there were specific areas of impairment in diagnosing and treating EDs, but all health professionals failed to recognize the role of family members in managing EDs.

The diagnosis of EDs was problematic for all health professionals, compared to psychiatrists. Early ED diagnosis is crucial to provide early care and promote better outcomes [3]. Individuals with EDs often require help for their somatic problems and access to physical health services more than to psychiatric services in the year preceding their ED diagnosis [25]. Thus, general practitioners, pediatricians and hospital doctors play an important role in diagnosis, and their difficulties with diagnosis can delay access to specialized care. This is supported by a multicenter European study showing that the cooccurrence of somatic symptoms at clinical referral is associated with a delay in access to specialized care [11]. The most frequent mistake among health professionals compared to psychiatrists is the inclusion of amenorrhea among the diagnostic criteria for AN, which may reflect the DSM-5 nosological change [22] and the need for continuing updates in medical education. It is remarkable that all health professionals, including psychiatrists, believe that blood tests can help rule out an ED diagnosis, outlining the excessive importance given to biological markers for diagnosis in psychiatry. In addition, another frequent mistake in the entire sample is that relatives of individuals with EDs should not be clearly informed by the doctors about the patient’s disorders: this is an important educational gap in light of the contribution that caregivers and family members give to ED diagnosis, access to specialist ED units and recovery [11, 26, 27].

The difficulties with diagnosis are similar to those that occurred when learning about risk factors and the psychopathology of EDs: this is the area with the highest number of items with correct answers reported by less than two thirds of the participants, regardless of their age. Low self-esteem, maladaptive perfectionism, and sensitivity to others’ judgments are not considered among the contributing factors to ED vulnerability, while eating behaviors of family members, having a family member suffering from an ED, drug abuse and having friendship with wrong or untrustworthy people are considered among the causes of EDs. On the other hand, respondents correctly acknowledged ED symptoms (e.g., binge-purging behaviors). This outlines an inadequate knowledge of ED etiopathogenesis [28, 29] and psychopathology that is at odds with the recent trend towards a reconceptualization of EDs as affective disorders with high importance of interpersonal problems [30–34]. Educational training should not only focus on the specific eating symptoms, but also on the processes that explain and promote ED symptoms [35]. This is true for all health professionals, including psychiatrists, who are more confident in certain areas (such as the causal role of family members, drug abuse and friendships) but not in all of them.

Treatment knowledge is another important area investigated in this study. Although psychiatrists are more confident in this area than other health professionals, they report the most frequent mistakes that occurred in the other groups: lack of effectiveness of psychotherapy, lack of knowledge of psychotherapeutic interventions specifically validated for EDs, consideration of the nutritional intervention as the first step in all individuals with EDs, and lack of the need to include family members in the treatment. These findings are in line with those suggesting that psychiatrists lack sufficient knowledge of both validated non-CBT psychotherapies for EDs and use of SSRIs in AN [19]. Evidence-based psychotherapies play a central role in the multidisciplinary approach to EDs [1]; thus, inadequate knowledge of psychotherapy interventions is a gap that needs to be addressed by educational programs. This aligns EDs to other psychiatric disorders where psychotherapy was found to be as effective as pharmacotherapy [36], supporting the need to disseminate psychotherapy effectiveness through educational training.

Treatment of EDs also includes handling physical complications: all health professionals performed well in this area, reporting errors for each item below 33%. It is noteworthy that all participants performed worse than psychiatrists, as non-mental health professionals should have a major role in diagnosing and treating physical complications.

Remarkably, surgeons were more stigmatized towards EDs than psychiatrists. The items that exhibited the most stigma were the following: assuming that individuals with EDs have chosen their abnormal eating behaviors; considering EDs not like any other illness; and making current and lifetime diagnoses based on the external physical appearance of the individual. However, no difference emerged for these items among the study groups. These findings should be considered in light of the observed impaired knowledge of EDs among clinicians and in the context of literature. Previous findings indicate that clinicians often perceive individuals with EDs responsible for causing their illness and prefer dealing with individuals with schizophrenia rather than those with EDs [37]. This is consistent with the more sympathetic attitudes that are observed towards illnesses that are believed to be ‘biologically’ caused, such as schizophrenia [37]. Overall, the present findings suggest that here may be a stigma against EDs that comes from health professionals and needs to be addressed in addition to the more well-known social stigma [38, 39].

Two general considerations are worth noting: first, better diagnosis and treatment knowledge was associated with older participants, suggesting that clinical experience may play a significant role in addition to educational training. Second, health professionals’ perception of family members is highly surprising: the importance of informing them about their relative’s diagnosis and including them in the treatment was often neglected, whereas they were often considered to a risk factor for EDs. This picture is in contrast with the increasing evidence that family-based treatments are the most effective in adolescents and young adults with EDs [40]. Educational programs should aim to inform clinicians that aiding caregivers in improving their responses to illness is crucial for recovery [41, 42].

Limitations of the study need to be acknowledged. First, the recruitment procedure, which included health professionals working in a unique university hospital, limits the generalizability of the study findings. Future studies are needed to determine if these findings are replicated in larger samples, including participants who work in different settings. Second, participants’ experience in the ED field was not evaluated, which could have had an impact on the study findings. Third, the reliability of subscores was not adequate: although we have partially faced this issue exploring the single item differences among the study groups, the validity of the hypothesized constructs of the questionnaire was not reliable in the present sample. Fourth, the identification of items in the questionnaire may be questionable: some items (namely, those referring to the management of physical complications) could refer to skills that exceed the required education for psychologists and do not represent a clinically significant gap, while others covering important area in the multidisciplinary approach to EDs (namely, the knowledge of severity criteria to define the level or the setting of care) have not been included. Likewise, the evaluation of diagnostic ability did not consider knowledge of diagnostic criteria: the primary focus was on the ability to identify ED signs and symptoms and their management as a general skill. Thus, conclusions about the ability to make early diagnosis must be interpreted with this limitation in mind.

## Conclusions

This is the first study to assess specific gaps in knowledge about the diagnosis, treatment and psychopathology of EDs among health professionals with different specializations. Compared to psychiatrists, other health professionals showed a certain degree of stigma towards EDs: these patients were seen as different from others and held responsible for their abnormal eating behaviors. In addition, inadequate knowledge of general psychopathology and risk factors associated with EDs, insufficient awareness of evidence-based psychotherapy for EDs and neglecting the central role that families play in promoting diagnosis and recovery are major gaps that needs to be addressed through education programs.

## Data Availability

The data that support the findings of this study are available from the corresponding author upon reasonable request.
